# Gut Microbial Composition and Predicted Functions Are Not Associated with Feather Pecking and Antagonistic Behavior in Laying Hens

**DOI:** 10.3390/life11030235

**Published:** 2021-03-12

**Authors:** Daniel Borda-Molina, Hanna Iffland, Markus Schmid, Regina Müller, Svenja Schad, Jana Seifert, Jens Tetens, Werner Bessei, Jörn Bennewitz, Amélia Camarinha-Silva

**Affiliations:** 1 Institute of Animal Science, University of Hohenheim, 70599 Stuttgart, Germany; danielbordam@gmail.com (D.B.-M.); hanna.iffland@uni-hohenheim.de (H.I.); markus_schmid@uni-hohenheim.de (M.S.); mregina96@aol.de (R.M.); schad.svenja@gmail.com (S.S.); jseifert@uni-hohenheim.de (J.S.); bessei@uni-hohenheim.de (W.B.); j.bennewitz@uni-hohenheim.de (J.B.); 2 Department of Animal Sciences, University of Göttingen, 37073 Göttingen, Germany; jens.tetens@uni-goettingen.de; 3 Center for Integrated Breeding Research, University of Göttingen, 37075 Göttingen, Germany

**Keywords:** gut microbiota, feather pecking, microbiability, laying hen, agonistic behavior

## Abstract

Background: Feather pecking is a well-known problem in layer flocks that causes animal welfare restrictions and contributes to economic losses. Birds’ gut microbiota has been linked to feather pecking. This study aims to characterize the microbial communities of two laying hen lines divergently selected for high (HFP) and low (LFP) feather pecking and investigates if the microbiota is associated with feather pecking or agonistic behavior. Methods: Besides phenotyping for the behavioral traits, microbial communities from the digesta and mucosa of the ileum and caeca were investigated using target amplicon sequencing and functional predictions. Microbiability was estimated with a microbial mixed linear model. Results: Ileum digesta showed an increase in the abundance of the genus *Lactobacillus* in LFP, while *Escherichia* was abundant in HFP hens. In the caeca digesta and mucosa of the LFP line were more abundant *Faecalibacterium* and *Blautia*. Tryptophan metabolism and lysine degradation were higher in both digesta and mucosa of the HFP hens. Linear models revealed that the two lines differ significantly in all behavior traits. Microbiabilities were close to zero and not significant in both lines and for all traits. Conclusions: Trait variation was not affected by the gut microbial composition in both selection lines.

## 1. Introduction

Feather pecking is a detrimental behavior pattern shown in layer flocks, leading to injured birds and, consequently to the welfare and economic problems. Research over the last few decades revealed the underlying mechanisms of feather pecking (for a review, see Rodenburg et al. [1]). Still, it remains an unsolved problem in the poultry industry worldwide. 

It is well known that environmental and genetic factors determine feather pecking. Previous research led to the assumption that the gut microbial composition is also involved in developing the undesired behavior. In laying hen lines divergently selected for feather pecking, supplementation with the essential amino acid L-tryptophan significantly reduced feather pecking by increasing the serotonergic tone [2]. Tryptophan supplementation increases the abundance of non-pathogenic bacteria (Bifidobacteria and Enterococci) known to support gut integrity and health [3]. A higher amount of feather pecking comes with a higher amount of feather eating [4,5,6,7], although raw feathers do not have any nutritional value [8]. Lutz et al. [9] identified a causal effect of feather eating on feather pecking using structural equation models. Meyer et al. [10] found differences in the gut microbiota and their metabolites between laying hen strains fed with different amounts of feathers. Some studies revealed that laying hen lines divergently selected for feather pecking also differed in some aspects of their gut microbial composition [11,12,13]. These findings suggested that the gut microbial composition might be associated with feather pecking and even might be one cause for it.

The ileum represents a major nutrient absorption site of the gastrointestinal (GI) tract in chickens and is dominated by *Lactobacillus* [14], *Streptococcus,* and *Escherichia coli* [15]. The caeca are colonized by a huge diversity of bacterial members, specifically Clostridiaceae, Bacteroidaceae, Lactobacillaceae, Proteobacteria, and butyrate-producing clusters as well as several uncultured bacteria [15,16]. The chicken caeca are an important fermentation site. They are responsible for the digestion of foods rich in cellulose, starch, and resistant polysaccharides, which impact the health and performance of the animals. Therefore, the contributing microbiota has been extensively examined [16,17,18]. Although it is known that the intestinal microbiota differs between mucosa and digesta samples, yet most studies characterized the digesta [19,20]. In all GI sections, mucosa samples showed higher microbial diversity than the digesta samples [15].

The term microbiability [21] describes the part of the phenotypic variance of a trait which is explained by the microbial composition. This parameter can be estimated with microbial mixed linear models. Microbiabilities in a medium-range were estimated for feed-related traits in pigs [22,23]. Verschuren et al. estimated high microbiabilities for the digestibility of several nutrients in fecal samples of pigs [24]. In a study on Japanese quails, medium microbiabilities for feed-related traits were identified [25]. Hence, the usefulness of microbiability to define the gut microbiome’s effect on feed-related traits in pigs and poultry could be revealed successfully.

Research on humans, rodents, and livestock showed that the gut microbiota composition influences behavior, e.g., anxiety-related, social, or feeding behavior [26]. Germ-free quail chicks were selected for high emotional reactivity (measured with tonic immobility test) and received either feces of conventional adults of the same line or a line selected for low emotional reactivity [27]. Germ-free chicks that received gut microbiota of the fearless line showed significantly less emotional reactivity than chicks with the fearful line’s microbiota. After two weeks, the gut microbial composition returned to its equilibrium, which was partially determined by the host genome [27]. Probiotic supplementation reduced fearfulness, improves memory, and reduces agonistic poultry behavior [26,28]. 

The present study aimed to characterize the gut microbial composition and its predicted functionality from two laying hen lines divergently selected for high (HFP) and low (LFP) feather pecking behavior. A possible influence of the gut microbiota composition toward feather pecking and agonistic behavior was investigated by applying microbial mixed linear models.

## 2. Materials and Methods

### 2.1. Birds and Experimental Procedures

The experiment and the experimental population’s establishments are described in Iffland et al. [29]. Briefly, hens of a White Leghorn layer strain were divergently selected for the severe form of feather pecking for 15 generations. Hens were reared together, regardless of the line, and were kept under the same conditions from hatching on. For behavioral observations at around 32 weeks of age, the hens were divided into smaller mixed HFP and LFP groups of about 40 animals and housed in deep litter pens. Observation, by experienced observers, began one week after group formation and took place during four consecutive days [30]. Due to a limited number of pens, two experimental runs were performed phenotyping a total of 492 hens (n_HFP_ = 270, n_LFP_ = 222). Besides others, three behavior traits were recorded, feather pecks delivered (FPD), aggressive pecks delivered (APD), and threats delivered (TD). The ethogram is displayed in Table 1.

All recorded traits were BoxCox transformed to reduce their deviation from a normal distribution. After the observation period of each experimental run, the hens were slaughtered at around 35 weeks of age. Both ileum and caeca were longitudinally opened, and digesta was collected with a sterile spoon. The mucosa was washed with a sterile phosphate-buffered saline solution and scraped with a sterile glass slide. Samples were stored in RNAlater at −80 °C until further analysis. The samples were divided into eight groups based on intestinal section (ileum or caecum), type of samples (digesta or mucosa), and line affiliation (HFP or LFP). The number of phenotyped animals with samples is shown separately for the sections and sample types in Table 2.

The German Ethical Commission of Animal Welfare of the State Government of Baden-Wuerttemberg, Germany approved the research protocol.

### 2.2. DNA Extraction Illumina Amplicon Sequencing and Bioinformatic Analysis

DNA was extracted from approximately 250 mg of each digesta and mucosa sample using FastDNA^TM^ SPIN Kit for soil from MP Biomedicals (Solon, OH, USA) following the manufacturer instructions. The quality and concentration of DNA were assessed through NanoDrop 2000 spectrophotometer (Thermo Scientific, Waltham, MA, USA), and DNA was stored until use at −20 °C. The V1-2 region of the 16S rRNA gene was amplified to produce the Illumina sequencing library. The protocol followed the methodology of Kaewtapee et al [31]. Briefly, one microliter of DNA was used as a template in the first PCR, where the forward primer contains a six-nucleotide barcode, and both primers have sequences complementary to the Illumina adapters. Master mixes include the PrimeSTAR^®^ HS DNA Polymerase kit (TaKaRa, Beijing, China). One microliter of the first PCR product was used in a second PCR following the same PCR conditions where both primers were complemented to the sequences of Illumina multiplexing and index primers. Amplicons were verified by agarose gel electrophoresis, purified, and normalized using SequalPrep Normalization Kit (Invitrogen Inc., Carlsbad, CA, USA). Samples and negative controls were sequenced using 250 bp paired-end sequencing chemistry on an Illumina MiSeq platform. 

Raw sequence reads obtained from Illumina MiSeq system (Illumina, Inc., San Diego, CA, USA) were analyzed using QIIME v1.9.1 pipeline [32], following a subsampled open-reference operational taxonomic units (OTUs) calling approach [33]. Demultiplexing and trimming of sequencing reads were done using the pipeline’s default parameters with a maximum sequence length of 360 bp [34]. The reads were merged into one FASTA-file and aligned using the SILVA Database (Release 132) [35]. Chimeras were identified and removed using usearch [36]. Reads were clustered at 97% identity into OTUs. Only OTUs present on average abundance higher than 0.0001% and a sequence length of >250 bp were considered for further analysis. The closest representative was manually identified with the seqmatch function of the Ribosomal Database Project. An average of 44,240 reads were obtained per sample. Sequences were submitted to European Nucleotide Archive under the accession number PRJEB40535. 

Prediction of functionality was carried out with the R package Tax4Fun2 [37], which relied on the SILVA database [38] and used the Kyoto Encyclopedia of Genes and Genomes (KEGG) hierarchy for the assignations, which comprise gene catalogs from sequenced genomes [39]. The biom table to assign this functionality was obtained from the QIIME pipeline. Genomes from 16S rRNA gene sequences identified in this study were downloaded from the National Center for Biotechnology Information (NCBI) database to produce a case-study-specific database for the ileum and caeca of laying hens.

### 2.3. Statistical Analysis

Linear discriminant analysis effect size (LEfSe) was applied to observe differences at the OTU level between the HFP and LFP line. The default cutoff was used, including *q* value < 0.1 and linear discriminant analysis score > 2.0 [40]. Random forest analysis overview was obtained at the OTU level to differentiate the impact of HFP and LFP on the prediction in microbiome data classification. Values by default were 500 trees, and the plots included the out-of-bag error [40].

Datasets were analyzed using PRIMER (version 7.0.9, PRIMER-E, Plymouth Marine Laboratory, Plymouth, UK) [41]. Data was standardized by total, and a similarity matrix was created using the Bray-Curtis coefficient [42]. PERMANOVA analysis, using a permutation method under a reduced model, was used to study the significant differences obtained when the dietary treatments were analyzed and considered significantly different if *p* < 0.05 [41]. The community similarity structure was depicted through non-metric multidimensional scaling plots. Similarity percentage analysis was used to identify the OTUs responsible for the groups’ differences. Diversity indices (Shannon diversity and Pielou’s evenness) were calculated based on abundance data with PRIMER software.

For estimation of the microbial variance components and the microbiability, the following microbial mixed linear model was applied using ASReml-R (Version 3.0) [43,44]. The model was applied separately for each trait and each gut section.

(1) y=Xb+m+e

where *y* is the vector containing the trait records for the corresponding trait (i.e., FPD, APD, or TD). *X* is a design matrix for vector *b*, which contained the line’s fixed effect, and a combination of experimental run and pen, if significant. Vector *e* denotes the random residual term. The residuals were modeled heterogeneously within the two feather pecking lines. The Vector *m* contains the random microbiota animal effects with distribution

(2) $$m\sim N\left(0,M\sigma_{m}^{2}\right)$$

with *M* being the microbial relationship matrix and $\sigma_{m}^{2}$ denoting the microbial variance. *M* was calculated as

(3) $$M=\frac{XX^{T}}{N}$$

with *N* being the number of OTUs, and *X* is a n × N matrix, where *n* is the number of animals. The standardized and log-transformed abundances of the OTUs are contained in *X* [22]. The microbiabilities $m_{l}^{2}$ for each trait and line *l* (*l* = HFP or LFP) were estimated as the fraction of the phenotypic variance in the lines explained by $\sigma_{m}^{2}$. A likelihood-ratio test on the random microbial animal effect was performed to test the significance of the microbiabilities. The test statistic was calculated as

(4) $$D=2\left[\log\left(L_{2}\right)-\log\left(L_{1}\right)\right]$$

with $L_{1}$ being the likelihood of the reduced model, i.e., model (1) without the random microbiota animal effect and $L_{2}$ the likelihood of the full model. The test statistic *D* under the null-hypothesis was chi-squared distributed with one degree of freedom. In addition, the two feather pecking lines were analyzed separately with the same model but without a fixed-line effect.

## 3. Results

### 3.1. Microbial Community

**Figure 1:**
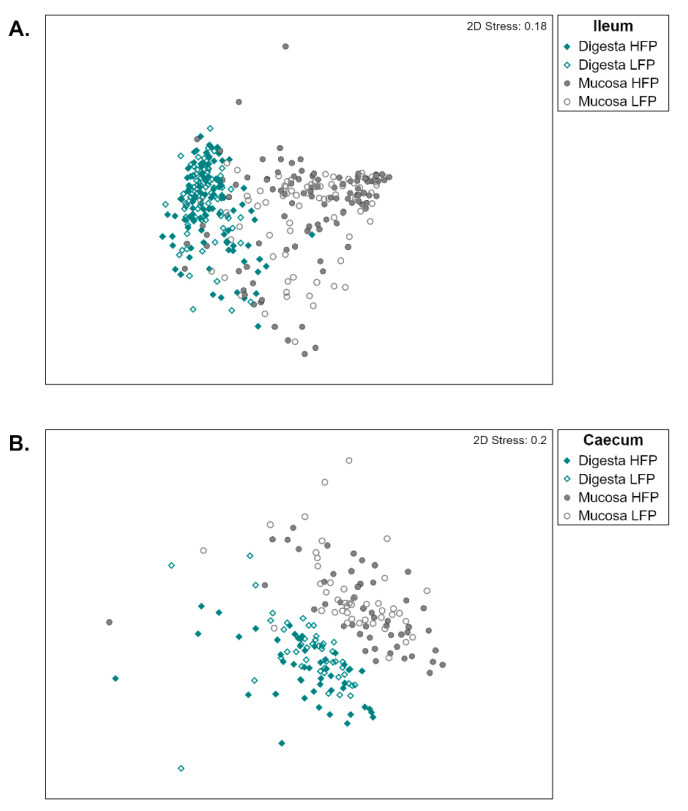
Non-metrical dimensional scaling plot showing the microbial community distribution for ileum (**A**) and caeca (**B**) samples of the high (HFP) and low (LFP) feather pecking line.

A significant (*p* = 0.003) difference in section and feather pecking line interaction was demonstrated by PERMANOVA (Table S1A). Samples of both ileum and caeca clustered by mucosa and digesta (Figure 1) and significant differences were obtained for the feather pecking lines and the type of samples (digesta or mucosa) (Table S1B,D,E). The Shannon diversity index showed significant (*p* ≤ 0.05) differences between ileum and caeca, being higher in the caeca but not between mucosa and digesta samples or the two lines of hens (Figure S1).

**Figure 2:**
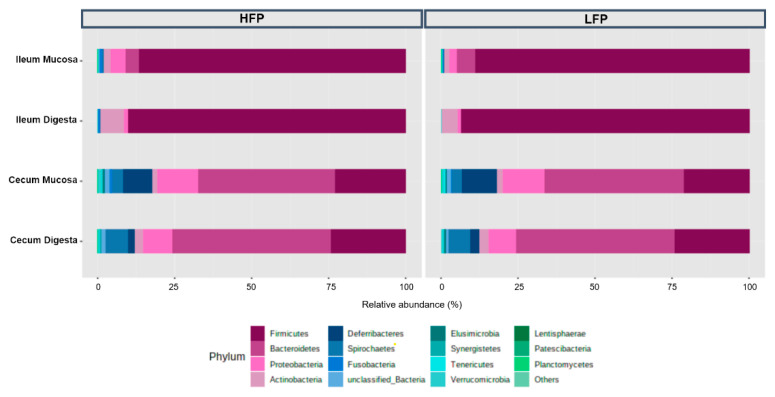
Percentage of relative abundance for phyla distribution in the ileum mucosa, ileum digesta, caeca mucosa, and caeca digesta in the high (HFP) and low (LFP) feather pecking line.

In the ileum digesta, the predominant phylum was Firmicutes with an average relative abundance of 93.5% for the LFP line, in comparison to 89.9% for the HFP line (*p* ≤ 0.05) (Figure 2). Actinobacteria were detected in the HFP line in higher abundance than in the LFP line (8.0% vs. 5.6%) (*p* ≤ 0.05). The percentage of Proteobacteria in the HFP line was also slightly higher (1.1%) than in the LFP line (0.8%). Ileum mucosa of LFP birds had more Firmicutes (88.6%) and Bacteroidetes (6.3%) than HFP birds (86.5% and 4.6%, respectively). Proteobacteria was more abundant in the HFP line (4.6%) than in the LFP line (2.2%) (*p* ≤ 0.05). Fusobacteria (*p* ≤ 0.05) and Actinobacteria were detected in higher relative abundance in HFP than LFP animals.

In caeca digesta samples, a significant (*p ≤* 0.05) difference was detected for Firmicutes relative abundance (24.2% in HFP compared to 23.9% in LFP). With a percentage lower than 3%, Deferribacteres and Tenericutes increased in HFP hens (*p* ≤ 0.05) (Figure 2). A significant (*p ≤* 0.05) difference was shown in caeca mucosa for Firmicutes (HFP 22.7% compared to LFP 21.1%). In LFP, Elusimicrobia and Fusobacteria were present in less than 2.5% relative abundance, but both increased in HFP hens (*p* ≤ 0.05) (Figure 2). Only Actinobacteria gave a higher value for the LFP birds (*p* ≤ 0.05). 

Random forest analysis was evaluated based on the global prediction error rate after 500 random forests [45]. After this classification, higher error rates for the microbial communities were obtained in the LFP line for ileum and caeca, mucosa and digesta (Figure S2). This result could imply a more predictable microbial composition in the HFP line since the lowest accuracy was observed in the LFP line. 

**Figure 3:**
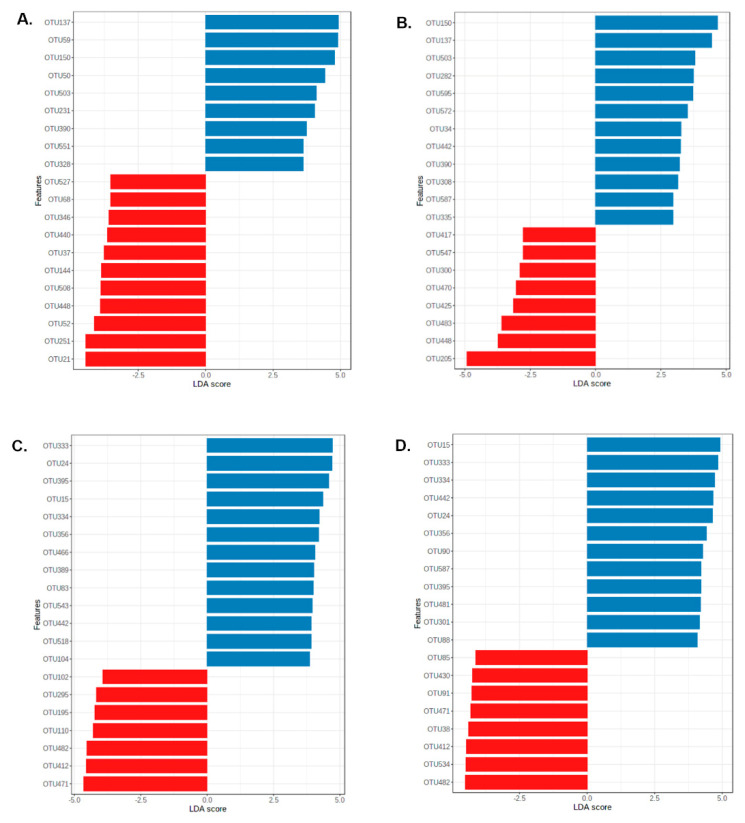
Linear discriminant analysis effect size (LEfSe) analysis for ileum digesta (**A**), ileum mucosa (**B**), caeca digesta (**C**), and caeca mucosa (**D**). The linear discriminant analysis (LDA) score is shown. The high feather pecking line is indicated by red and the low feather pecking line by blue.

**Figure 4:**
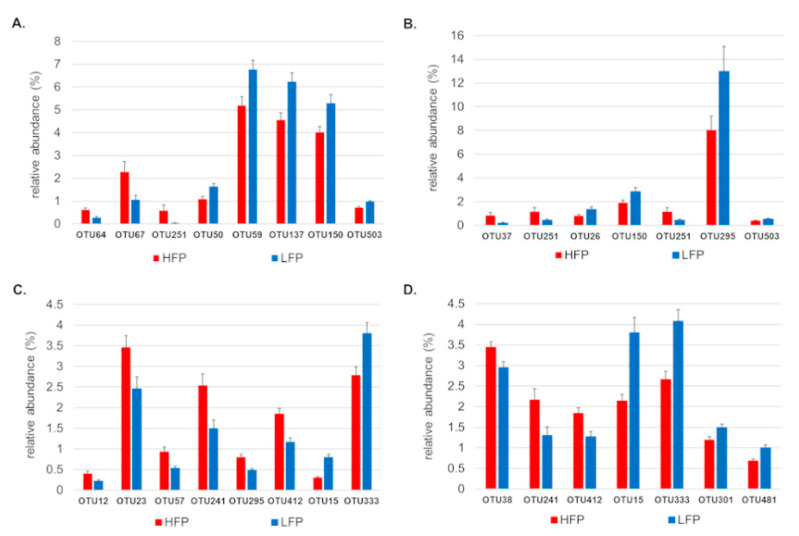
Relative abundance for the OTUs showing a significant difference for ileum digesta (**A**), ileum mucosa (**B**), caeca digesta (**C**), and caeca mucosa (**D**). The high (HFP) feather pecking line is indicated by red and the low (LFP) feather pecking line by blue.

LefSe analysis was consistent, showing differences for the same OTUs in the ileum and caeca of the two feather pecking lines (Figure 3). *Lactobacillus* species (OTUs: 50, 59, 137, 150, 231, 390, 503, 551) based on LefSe analysis only appeared in the ileum and the occurrence was higher in the LFP line (Figure 3A,B). In the ileum digesta, the relative abundance of the OTUs 64 (Unclassified (Unc.) *Olsenella*), 67 (Unc. Clostridiaceae 1), and 251 (*Clostridium rectum*) were higher in the HFP line than in the LFP line (Figure 4). Microbial communities in the HFP hens for the ileum mucosa also included OTU37 (*Escherichia coli*) and OTU251 (*p* ≤ 0.05) (Figure 4).

The caeca were colonized by a greater number of bacterial species than the ileum as also represented by a higher diversity index (Figure S1). OTU12 (*Lactobacillus kitasatonis*), OTU23 (Unc. *Paraprevotella*), OTU57 (*Lactobacillus gallinarum*), OTU241 (Unc. Bacteroidales), OTU295 (Unc. *Romboutsia*), and OTU412 (Unc. Proteobacteria) (*p* ≤ 0.05) (Figure 4) were detected in higher relative abundance in the HFP line. Less OTUs resulting in significant (*p* ≤ 0.05) differences were observed for the LFP line; the OTU15 (Unc. *Mucispirillum*) and OTU333 (Unc. Bacteroidaceae) had higher abundances (Figure 4). In the caecum mucosa of HFP line, OTU38 (Unc. *Phascolarctobacterium*), OTU241 (Unc. Bacteroidales), and OTU412 (Unc. Proteobacteria) were more abundant (*p* ≤ 0.05); while for the LFP line again OTU15, OTU 333, and OTU301 (Unc. *Suterella*) and OTU481 (Unc. *Treponema*) (*p* ≤ 0.05) were detected (Figure 4).

Functional prediction showed significant differences for the feather pecking lines in the caeca microbiota, but not in the ileum (Table S2B–E). In the category of amino acid metabolism, tryptophan metabolism, and lysine degradation appeared in both digesta and mucosa, and it was higher in the HFP line. In contrast, cysteine and methionine metabolism and lysine biosynthesis were only predicted in the digesta with increased values in the HFP line. Metabolic pathways of other amino acids were observed in increased abundance in the LFP line in both the digesta and mucosa samples (Figure S3).

In the category of carbohydrate metabolism, 10 out of 15 subcategories resulted in a significant (*p* ≤ 0.05) difference between the digesta samples of both lines. At the same time, only five were found in the mucosa (Figure S4). In both sections, glycolysis/gluconeogenesis and amino sugar and nucleotide sugar metabolism were higher in the HFP line. LFP hens had more functions related to glyoxylate and dicarboxylate metabolism and C5-branched dibasic acid metabolism. The category of energy metabolism showed enhanced numbers of nitrogen metabolism in LFP digesta and mucosa samples. Oxidative phosphorylation and carbon fixation pathways were only observed in the digesta and enhanced for the LFP line (Figure S5). Membrane transports had higher values for the bacterial secretion system subcategory in LFP birds. ATP-binding cassette (ABC) transporters and phosphotransferase system increased in the HFP birds (Figure S6). LFP birds (digesta and mucosa) showed major significant differences (Figure S7) regarding biosynthesis of secondary metabolites.

Lipid metabolism increased in both digesta and mucosa samples of HFP line for glycerolipid, arachidonic acid, and glycerophospholipid metabolism (*p* ≤ 0.05) (Figure S8). Cell motility, specifically biofilm formation in *E. coli* and *Pseudomonas aeruginosa,* was predicted in the HFP samples (Figure S9).

### 3.2. Microbial Parameters 

The linear models revealed that the lines differ significantly in the three behavior traits in all subsets of animals. The estimations of microbial parameters and microbiabilities for ileum mucosa in the HFP and LFP lines are shown in Table 3. For the agonistic traits APD and TD, low to medium microbial animal effects were estimated, which resulted in low to medium microbiabilities without significance. For FPD in ileum mucosa and all three traits in the other intestinal sections and samples, i.e., ileum digesta, caecum mucosa, and caecum digesta, the microbial animal effect estimators were fixed at the boundary by the algorithm. Hence, the microbial animal effects and thus the microbiabilities were nearly zero and not significant.

The results of the separated analyzes of the two lines (not shown) revealed that none of the microbial animal effects in any of the lines and traits were significant.

## 4. Discussion

### 4.1. Microbial Community

The characterization of the intestinal microbiota of both lines used in this study resulted in a similar microbial composition as previously described in laying hens [13] and chickens [15,16], including specific patterns such as the higher diversity in the caeca compared to the ileum. *Lactobacillus* species are known to be essential inhabitants of the GI tract of animals and are used as probiotic microorganisms due to their health-promoting properties [46,47]. *Lactobacillus* reduces the GI colonization of pathogens in broiler chickens such as *Campylobacter* [48], *Clostridium* [49], and *Salmonella* [50]. LEfSe analysis showed that in the ileum of LFP laying hens, mainly *Lactobacillus* species, such as *L. johnsonii* and *L. crispatus*, drove the community. La Ragione et al. [49] found that *L. johnsonii* significantly reduced *E. coli* colonization in chickens’ small intestine. *L. crispatus* showed high amylase activity, positively affecting feed conversion and broiler performance [51]. *Lactobacillus* stimulated serotonin receptors [52] or increased serotonin and dopamine in the brain [53], influencing the locomotor activity or decreased anxiety and depression-related behavior [53,54,55]. 

The role of *Romboutsia* species in the small intestines is still unknown due to the limited availability of cultivated representatives [56]. Here, this genus was highly dominating the caeca digesta of HFP birds. The genus *Mucispirillum* was positively associated with mucus production [57] and therefore related with a healthy intestine [58,59], in the present study it was detected in higher abundance in LFP than in HFP hens.

Random forest analysis is intended to classify and select the microbial data’s main features [40]. It demonstrated that the HFP line comprises less out-of-bag error, which probably indicates a specific microbiota simpler to predict. In contrast, LFP promotes a host-microbiome with more differences leading to higher misclassification rates [45].

In the literature, it was shown that birds fed with feathers differed from control birds in the microbial metabolites and microbial composition. Feather fed birds showed higher numbers of enterobacteria in the ileum and caecum and higher numbers of clostridia in the caecum [10]. Thus, it is expected that feathers’ consumption could change the microbial composition [13] and is assisted by the identified appearance of *E. coli* in LEfSe analysis in ileum digesta of the HFP hens. 

A previous study demonstrated that gut microbes thrive the release of metabolites such as hydrogen sulfide and other sulfur-containing substances or biogenic amines, which are reactive and potentially influence behavior [10]. These findings were also observed in the predicted functions from this study. Another potential influence on behavior was the predicted promotion of biosynthesis of tryptophan in LFP hens. Tryptophan is the precursor of serotonin, and it was assumed that the alteration on the serotonergic system would impact the feather pecking behavior [60]. Indeed, feather pecking was reduced in diets with 2% of tryptophan compared to supplementation of 0.16% [2,60].

### 4.2. Microbial Parameters

For some of the traits, sample types, and gut sections as for FPD in Table 1, no variance components could be estimated. This was in line with the clustering of the microbial community distribution shown in Figure 1A. Except for ileum mucosa, no cluster separation was observed within and between the lines. For ileum mucosa, a tendency of separation of the two lines was noticeable implying a differentiation of the two lines’ gut microbiota. The limited number of individuals in the present study might be the reason variance components could only be estimated in the ileum mucosa when both lines were analyzed together. No significant effect was determined in the estimated variance components and microbiabilities. Thus, for the behavior traits FPD, APD, and TD, no part of the phenotypic variance could be associated with the gut microbial composition. This means that even though the hens differed significantly in these behavior traits as well as in some fractions of the gut microbial composition, the gut microbiota composition was not associated with the behavior traits. The two feather pecking lines of the 15th generation were genetically distinguishable from each other with huge allele frequency differences between the two lines. This resulted in a mean F_ST_ value of 0.16 [30], which was predominately due to drift and only to a minor extent due to selection [30,61]. Hence, these genetic differences might be the cause for the microbial differences as it is known that the microbiota is partially shaped by the host genome [62]. Another explanation might be that HFP hens picked and digested more feathers than LFP hens which altered the gut microbial composition [10]. 

Besides the idea to repeat the study with larger cohorts, one might apply a similar experimental setup as Kraimi et al. [27], where a microbiota transfer between divergently selected feather pecking lines was conducted, to finally rule out whether the microbiota is responsible for the differences in feather pecking behavior. This setup would also include gut microbiota, which cannot be identified or cultivated with the current techniques. Hence, if there is any influence of the microbiota on feather pecking, it could be revealed by this experiment.

## 5. Conclusions—Does the Microbial Composition in Ileum or Caecum Influences Feather Pecking Behavior?

No, as far as it is known from the recent results. Although significant differences in the gut microbial composition between the HFP and LFP line were found, it was impossible to show the microbiome’s influence on the behavior traits FPD, APD, and TD.

## Figures and Tables

**Table 1:** Ethograms of the recorded traits feather pecks delivered (FPD), aggressive pecks delivered (APD) and threats delivered (TD).

Trait	Definition
FPD	Non-aggressive severe pecks or pulls are directed to the plumage of conspecifics, sometimes resulting in pulled-out feathers and a recipient, which tolerates or moves away. Therefore, the deliverer does not adopt any special body posture.
APD	Pecks delivered in an upright body posture against (mainly) the head and other parts of the recipient’s body.
TD	Visual fixation on the recipient in an upright body posture followed by the recipient’s avoidance or withdrawal behavior.

**Table 2:** Number of animals of the high (HFP) and low (LFP) feather pecking line with samples for the respective gut section and type of samples used in the microbial linear mixed model.

Gut Section and Sample Type	HFP	LFP	∑
Ileum mucosa	96	73	169
Ileum digesta	95	82	177
Caecum mucosa	48	42	90
Caecum digesta	48	43	91

**Table 3:** Estimated microbial parameters for the ileum mucosa microbial composition of 169 hens of the high (HFP) and low (LFP) feather pecking line for the three behavior traits feather pecks delivered (FPD), aggressive pecks delivered (APD) and threats delivered (TD).

Ileum Mucosa						
	$\sigma_{m}^{2}\;(\mathrm{SE})$	$\sigma_{e}^{2}\;(\mathrm{HFP})\;(\mathrm{SE})$	$\sigma_{e}^{2}\;(\mathrm{LFP})\;(\mathrm{SE})$	$m_{HFP}^{2}$	$m_{LFP}^{2}$	*p*-Value
FPD	<0.001 (NA)	0.55 (0.08)	0.26 (0.05)	<0.001	<0.001	1
APD	0.08 (0.11)	1.04 (0.17)	0.52 (0.12)	0.07	0.13	0.54
TD	0.19 (0.12)	1.04 (0.17)	0.35 (0.10)	0.15	0.35	0.37

## Data Availability

Sequences were submitted to European Nucleotide Archive under the accession number PRJEB40535.

## Supplementary Materials

The following are available online at https://www.mdpi.com/2075-1729/11/3/235/s1, Figure S1. Shannon diversity index for the ileum and caeca in the mucosa and digesta samples coming from the high (HFP) and low feather pecking (LFP) laying hen lines. Figure S2. Random forest analysis based on the estimation for the out of the error bag (OOB) (y-axis) with a bootstrap of 500 created trees (x-axis), based on abundance information of operational taxonomic units at genus level data in ileum digesta (A), ileum mucosa (B), caeca digesta (C), and caeca mucosa (D). The table explained the classification performance for the high feather pecking line (green), the low feather pecking line (blue), and across both lines (red). Figure S3. Functional predictions for the caeca digesta and mucosa in the subcategory amino acid metabolism in the high and low feather pecking laying hen lines. Figure S4. Functional predictions for the caeca digesta and mucosa in the subcategory carbohydrate metabolism in the high and low feather pecking laying hen lines. Figure S5. Functional predictions for the caeca digesta and mucosa in the subcategory energy metabolism in the high and low feather pecking laying hen lines. Figure S6. Functional predictions for the caeca digesta and mucosa in the subcategory membrane transport in the high and low feather pecking laying hen lines. Figure S7. Functional predictions for the caeca digesta and mucosa in the subcategory biosynthesis of other secondary metabolites in the high and low feather pecking laying hen lines. Figure S8. Functional predictions for the caeca digesta and mucosa in the subcategory lipid metabolism in the high and low feather pecking laying hen lines. Figure S9. Functional predictions for the caeca digesta and mucosa in the subcategory cell motility in the high and low feather pecking laying hen lines. Table S1A–E. Permanova test for the 16S rRNA gene identified bacterial species dataset obtained from the gut microbiome samples of the mucosa and digesta (type) taken either from the ileum or caeca (section) from the high and low feather pecking laying hen lines (line). Table S2A–E. Permanova test for the predicted functions based on 16S rRNA gene identified bacterial species obtained from the gut microbiome samples of the mucosa and digesta (type) taken either from the ileum or caeca (section) from the high and low feather pecking laying hen lines (line).
