# Supplementary material for: Gut Microbial Composition and Predicted Functions Are Not Associated with Feather Pecking and Antagonistic Behavior in Laying Hens
Source: Life (Basel). 2021 Mar 12;11(3):235. doi: 10.3390/life11030235 (PMC8001194; doi:10.3390/life11030235)
Supplement: Supplementary file 1 [file life-11-00235-s001.pdf]

Gut Microbial Composition and Predicted Functions are not Associated with Feather Pecking and Antagonistic Behavior in Laying Hens

Daniel Borda-Molina[1,+], Hanna Iffland[1,+], Markus Schmid[1], Regina Müller[1], Svenja Schad[1], Jana Seifert[1], Jens Tetens[2,3], Werner Bessei[1], Jörn Bennewitz[1] and Amélia Camarinha-Silva[1,*]

Supplementary Material

1 Supplementary Figures

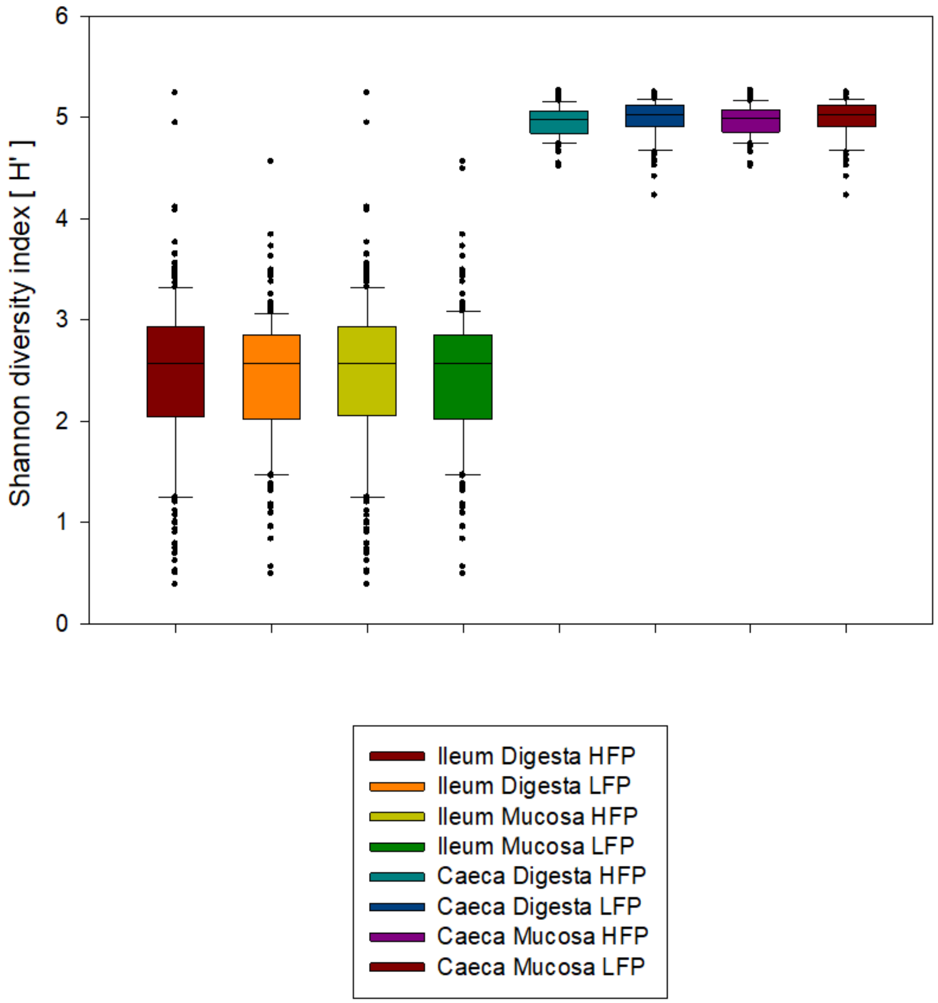

Figure 1. Shannon diversity index for the ileum and caeca in the mucosa and digesta samples coming from the high (HFP) and low feather pecking (LFP) laying hen lines.

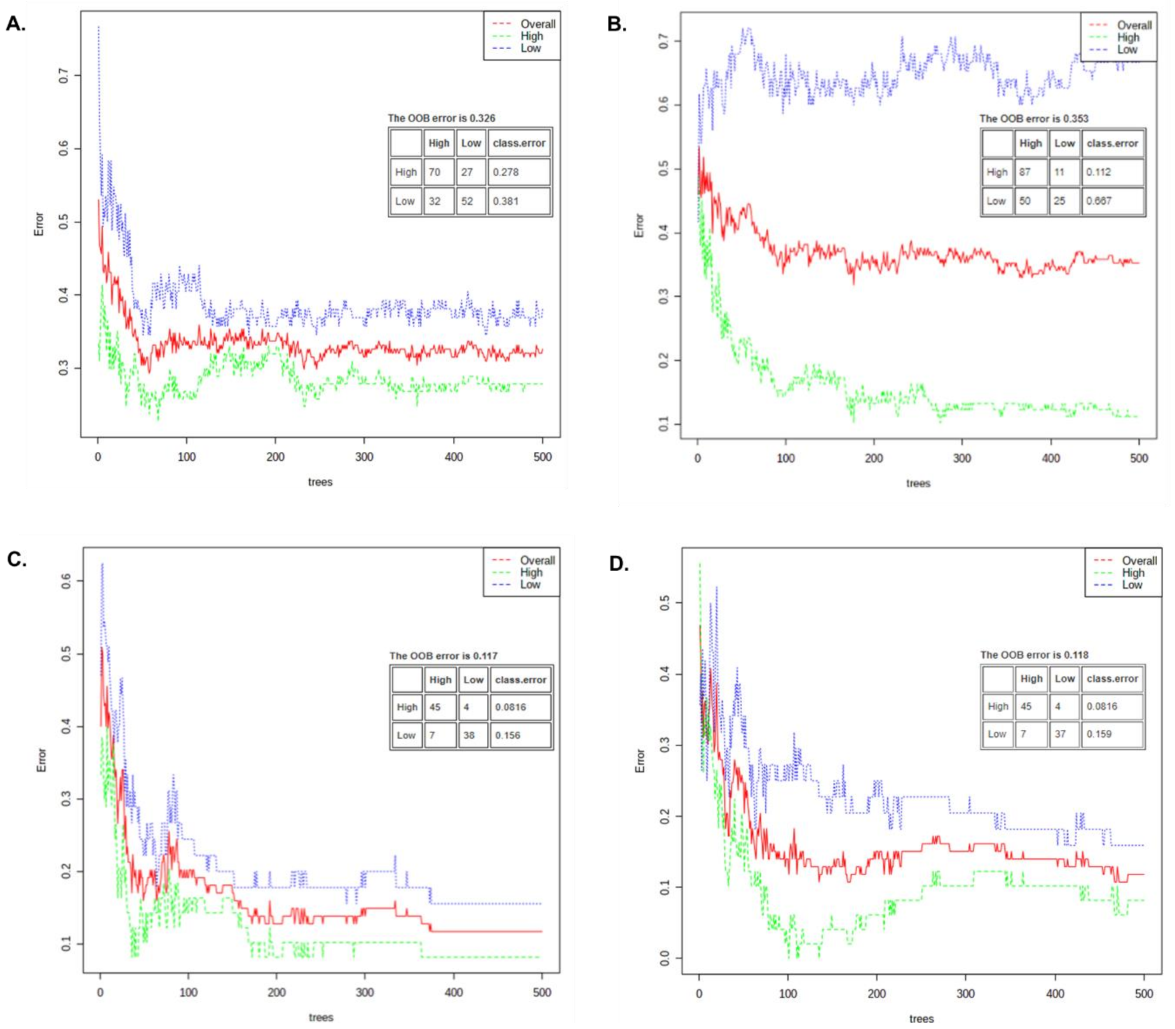

Figure S2. Random forest analysis based on the estimation for the out of the error bag (OOB) (y-*axis*) with a bootstrap of 500 created trees (x-*axis*), based on abundance information of operational taxonomic units at genus level data in ileum digesta (A), ileum mucosa (B), caeca digesta (C), and caeca mucosa (D). The table explained the classification performance for the high feather pecking line (green), the low feather pecking line (blue), and across both lines (red).

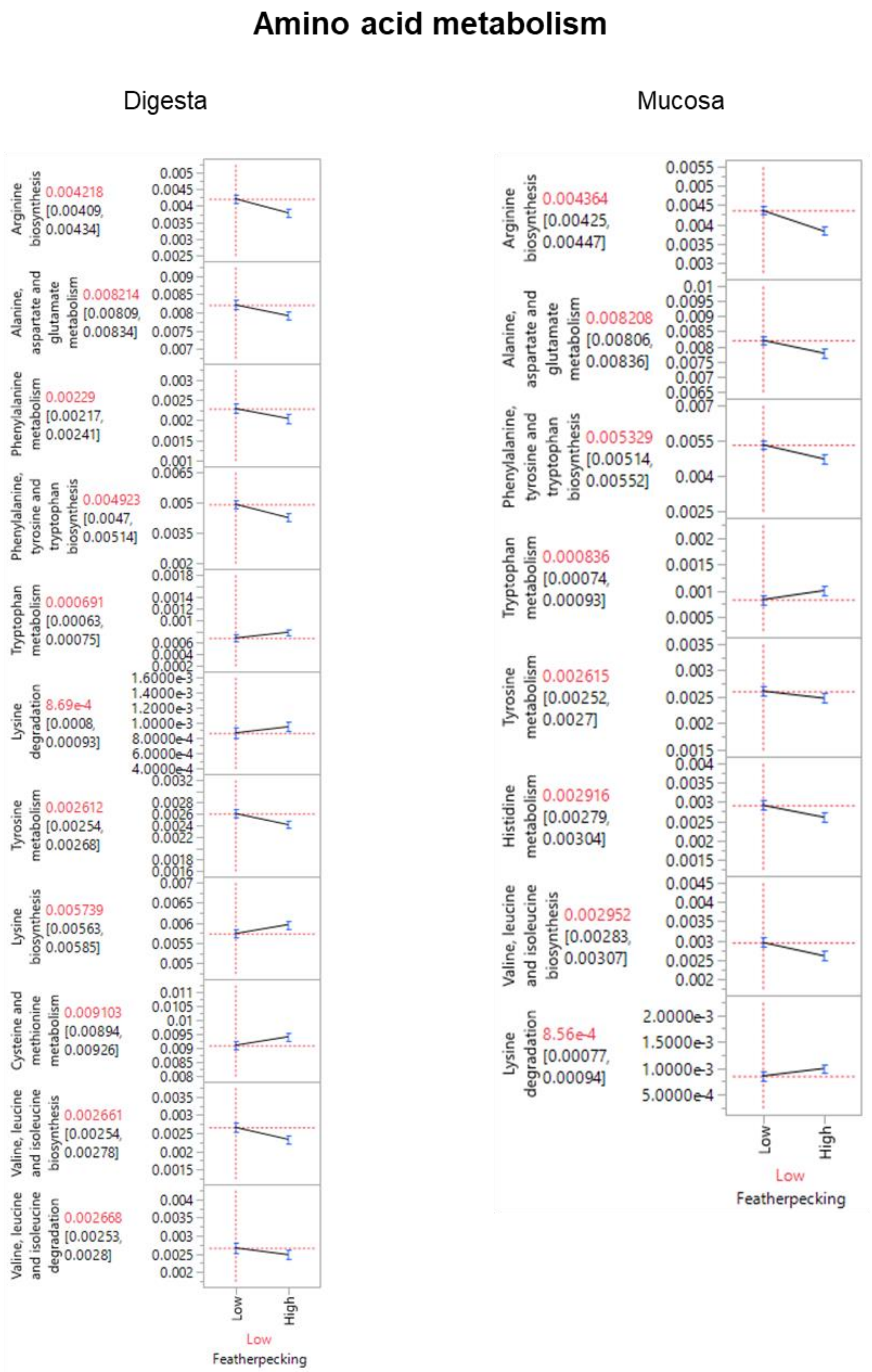

Figure S3. Functional predictions for the caeca digesta and mucosa in the subcategory amino acid metabolism in the high and low feather pecking laying hen lines. The mean values of the measures are highlighted in red. The range of the observed values is given in brackets.

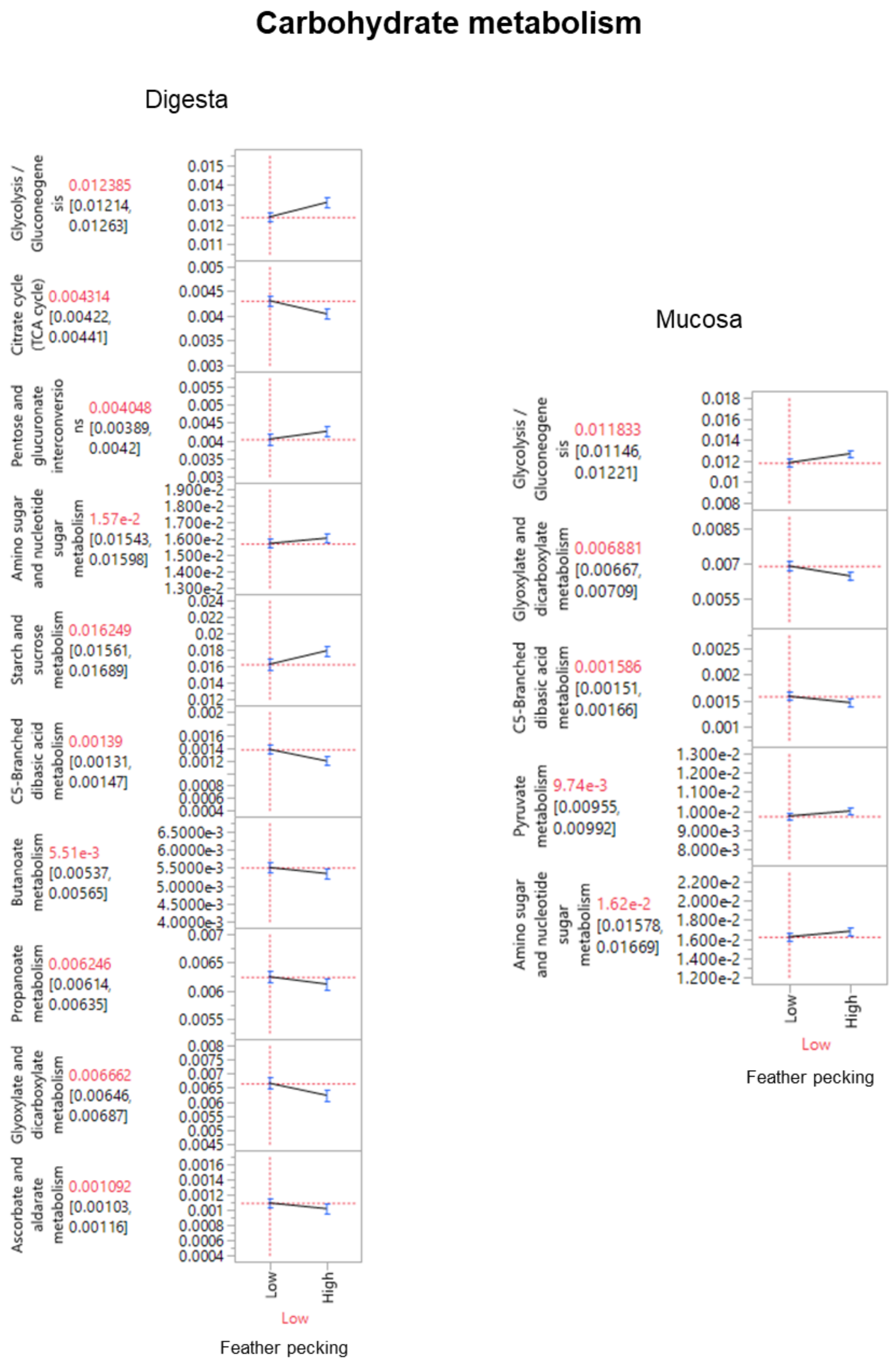

Figure S4. Functional predictions for the caeca digesta and mucosa in the subcategory carbohydrate metabolism in the high and low feather pecking laying hen lines. The mean values of the measures are highlighted in red. The range of the observed values is given in brackets.

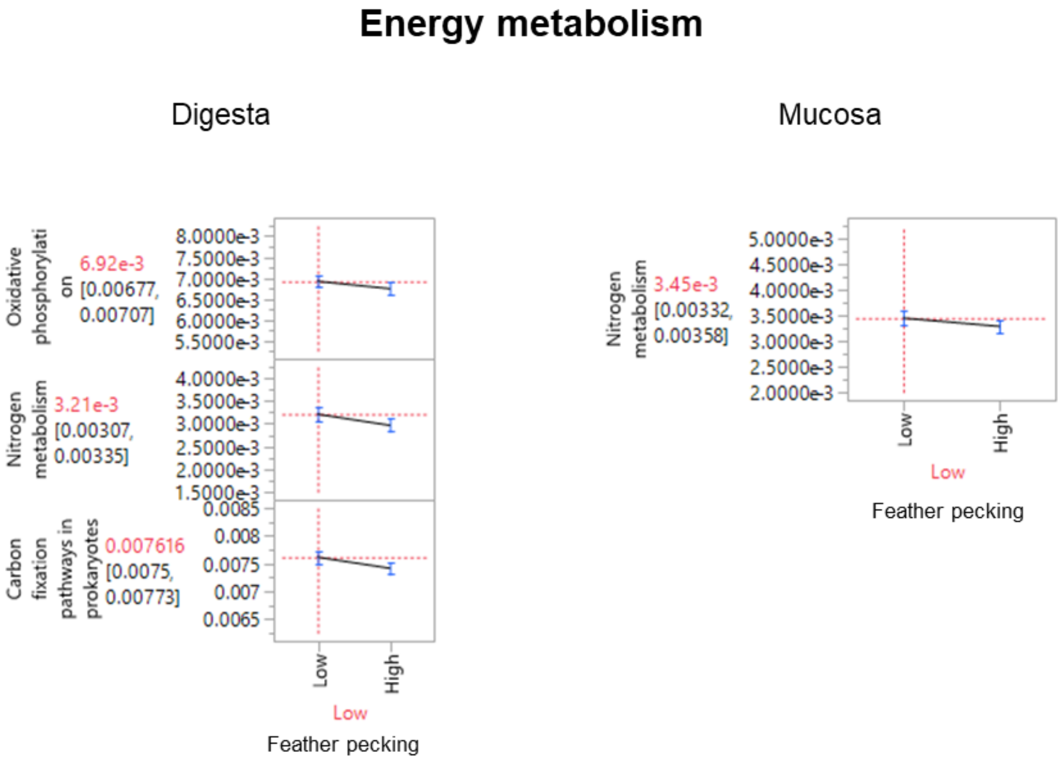

Figure S5. Functional predictions for the caeca digesta and mucosa in the subcategory energy metabolism in the high and low feather pecking laying hen lines. The mean values of the measures are highlighted in red. The range of the observed values is given in brackets.

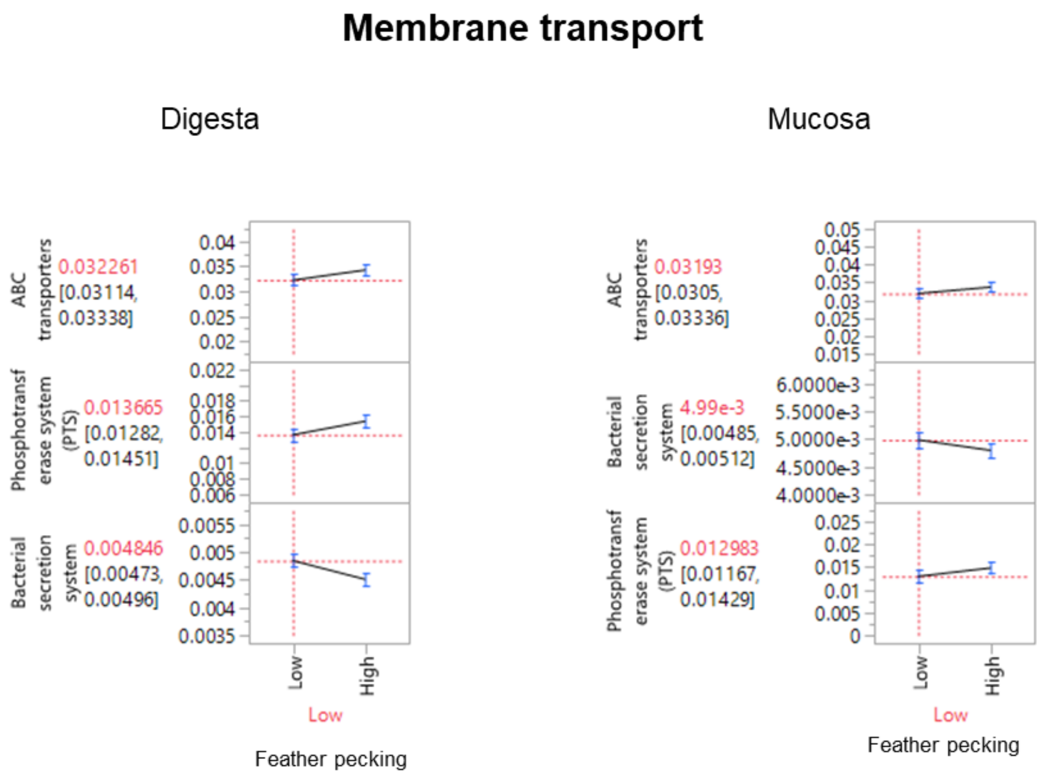

Figure S6. Functional predictions for the caeca digesta and mucosa in the subcategory membrane transport in the high and low feather pecking laying hen lines. The mean values of the measures are highlighted in red. The range of the observed values is given in brackets.

Biosynthesis of other secondary metabolites

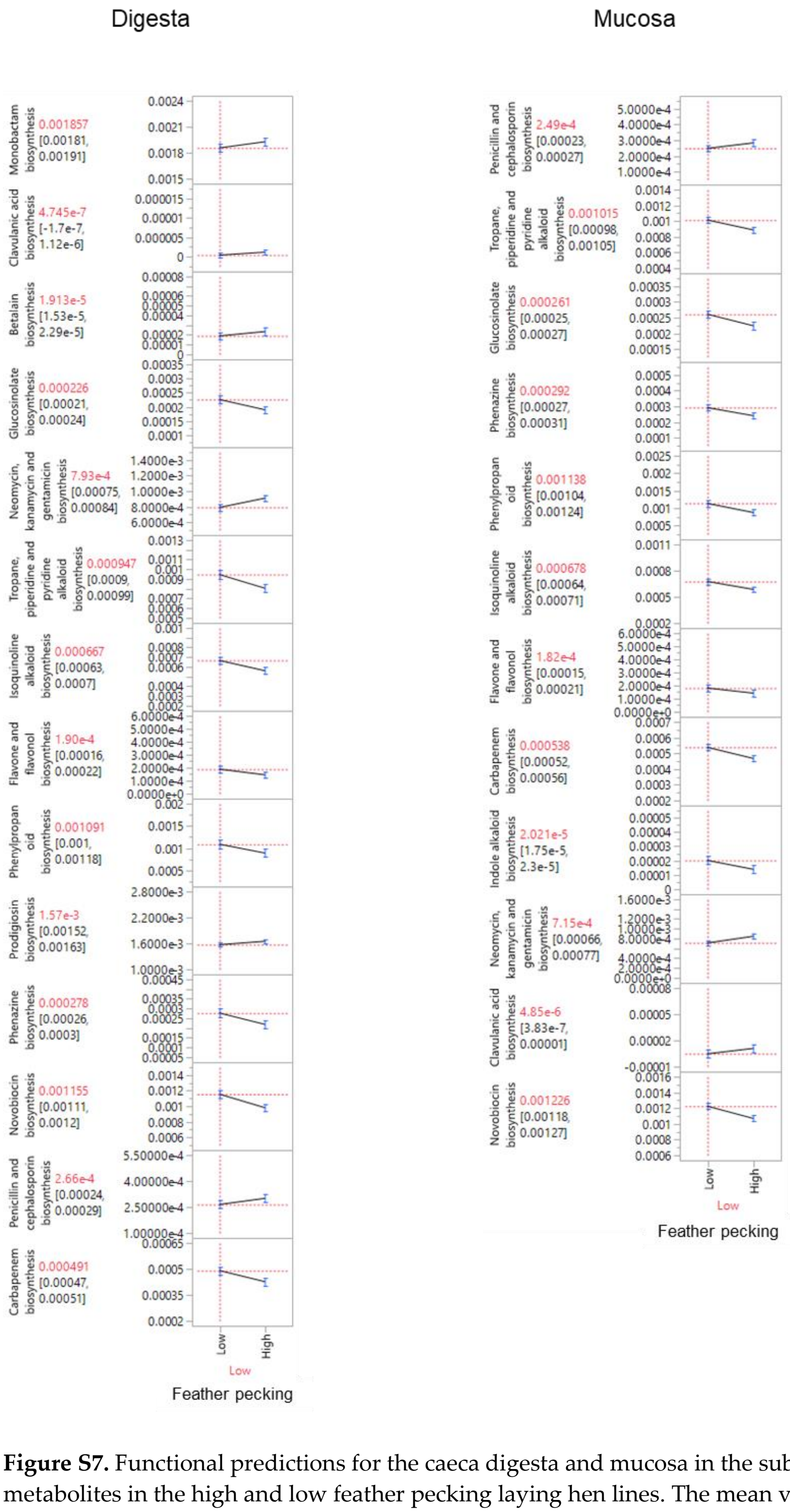

Figure S7. Functional predictions for the caeca digesta and mucosa in the subcategory biosynthesis of other secondary metabolites in the high and low feather pecking laying hen lines. The mean values of the measures are highlighted in red. The range of the observed values is given in brackets.

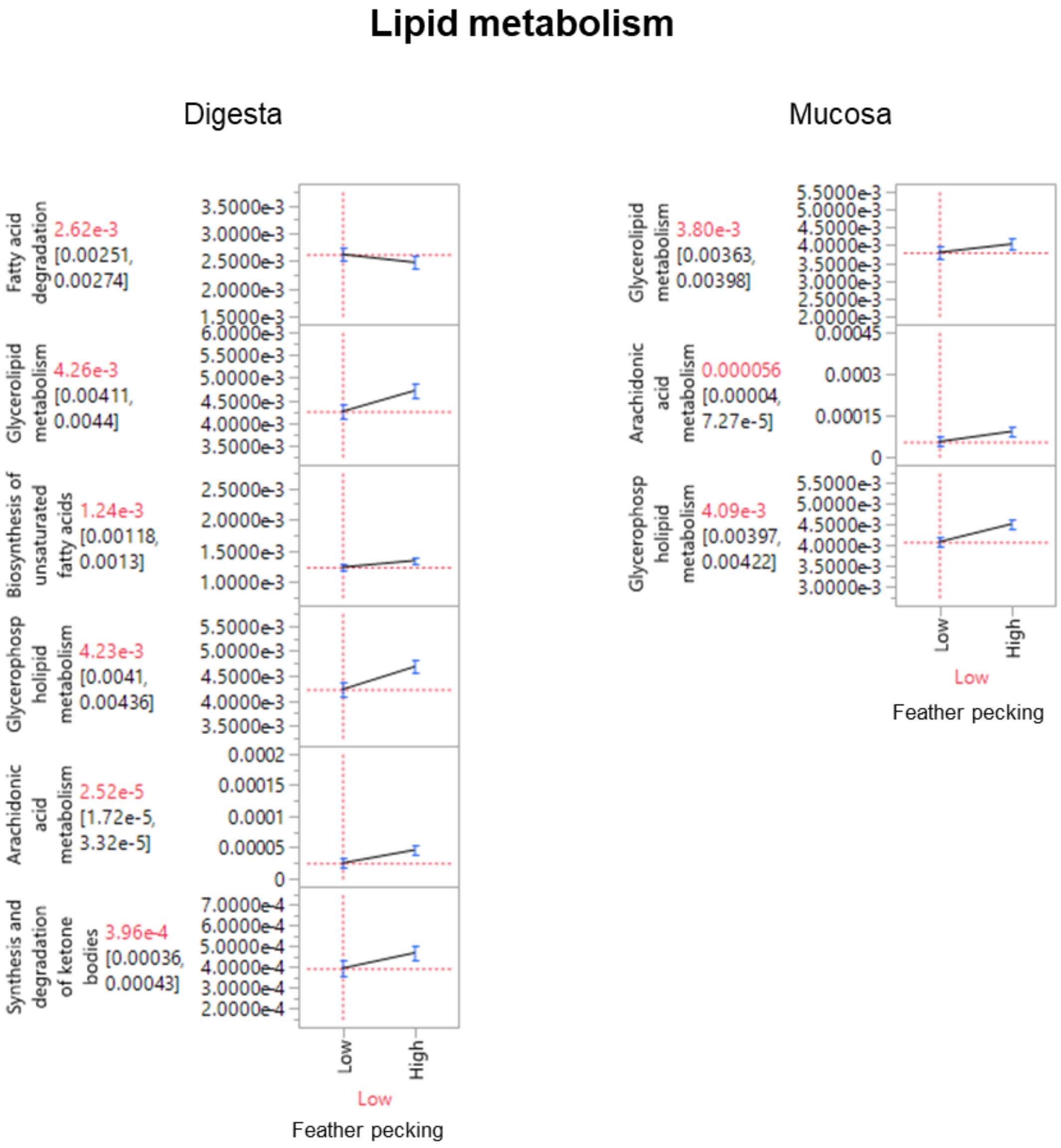

Figure S8. Functional predictions for the caeca digesta and mucosa in the subcategory lipid metabolism in the high and low feather pecking laying hen lines. The mean values of the measures are highlighted in red. The range of the observed values is given in brackets.

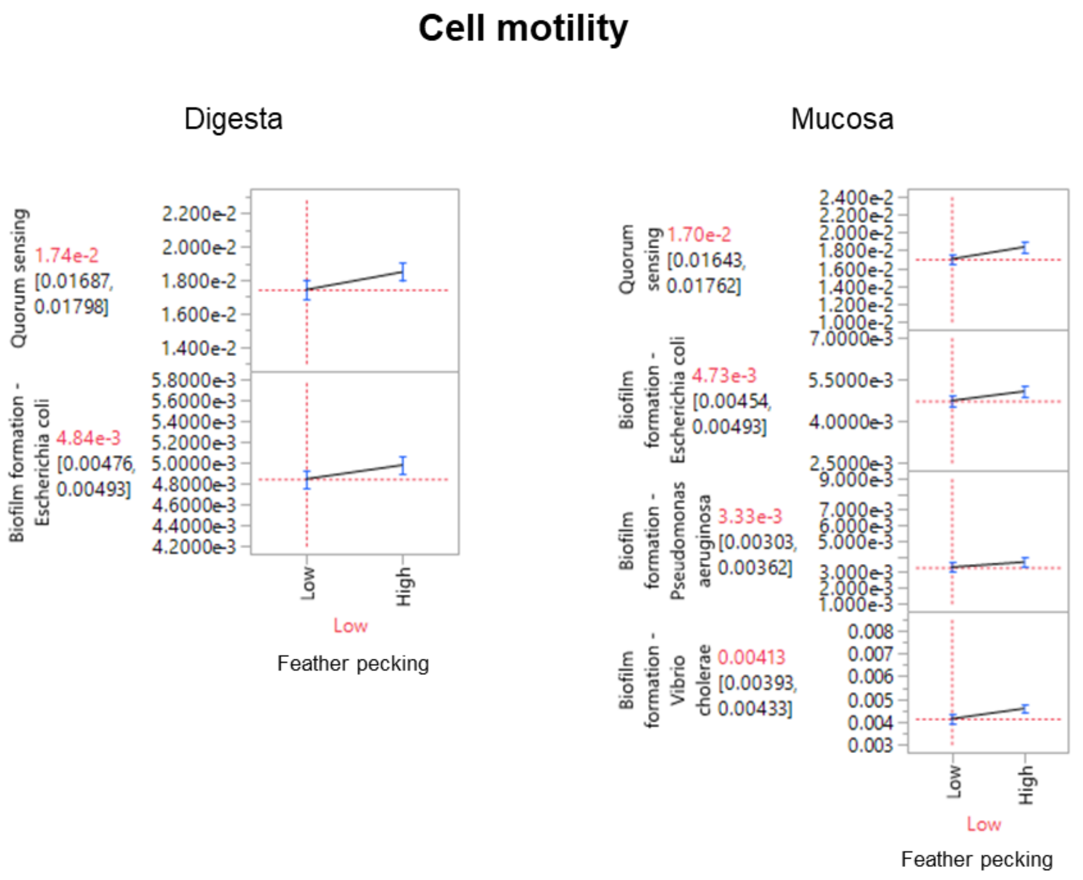

Figure S9. Functional predictions for the caeca digesta and mucosa in the subcategory cell motility in the high and low feather pecking laying hen lines. The mean values of the measures are highlighted in red. The range of the observed values is given in brackets.

2 Supplementary Tables

Table 1. A-E. Permanova test for the 16S rRNA gene identified bacterial species dataset obtained from the gut microbiome samples of the mucosa and digesta (type) taken either from the ileum or caeca (section) from the high and low feather pecking laying hen lines (line).

A. Overall test

**PERMANOVA table of results**						
**Source**	**df**	**SS**	**MS**	**Pseudo-F**	**P(perm)**	**Unique perms**
**Type**	1	89289	89289	66.759	0.0001	9931
**Section**	1	6.95E+09	6.95E+09	519.66	0.0001	9936
**Line**	1	5954.3	5954.3	44.519	0.0033	9938
**Type x Section**	1	83185	83185	62.195	0.0001	9935
**Type x Line**	1	2197.4	2197.4	1.643	0.1369	9924
**Section x Line**	1	6061.5	6061.5	45.321	0.0033	9940
**Type x Section x Line**	1	1981.8	1981.8	14.817	0.171	9936
**Residual**	533	7.13E+09	1337.5			
**Total**	540	1.65E+09				

B. Ileum digesta

PERMANOVA table of results						
**Source**	**df**	**SS**	**MS**	**Pseudo-F**	**P(perm)**	**Unique perms**
**Line**	1	4002.1	4002.1	3.1189	0.0046	9937
**Residual**	179	2.2968E5	1283.2			
**Total**	180	2.33E+05				

C. Ileum mucosa

PERMANOVA table of results						
**Source**	**df**	**SS**	**MS**	**Pseudo-F**	**P(perm)**	**Unique perms**
**Line**	1	2803.1	2803.1	1.4978	0.1657	9945
**Residual**	171	3.2003E5	1871.5			
**Total**	172	3.2283E5				

D. Caecum digesta

PERMANOVA table of results						
**Source**	**df**	**SS**	**MS**	**Pseudo-F**	**P(perm)**	**Unique perms**
**Line**	1	4087.4	4087.4	4.8409	0.0001	9903
**Residual**	92	77680	844.35			
**Total**	93	81768				

E. Caecum mucosa

PERMANOVA table of results						
**Source**	**df**	**SS**	**MS**	**Pseudo-F**	**P(perm)**	**Unique perms**
**Line**	1	4714.3	4714.3	5.0187	0.0001	9895
**Residual**	91	85480	939.34			
**Total**	92	90194				

Table 2. A-E. Permanova test for the predicted functions based on 16S rRNA gene identified bacterial species obtained from the gut microbiome samples of the mucosa and digesta (type) taken either from the ileum or caeca (section) from the high and low feather pecking laying hen lines (line).

A. Overall test

PERMANOVA table of results						
**Source**	**df**	**SS**	**MS**	**Pseudo-F**	**P(perm)**	**Unique perms**
**Type**	1	70.081	70.081	3.9665	0.0147	9940
**Section**	1	464	464	26.262	0.0001	9948
**Line**	1	5130.5	5130.5	290.38	0.0001	9930
**Type x Section**	1	13.447	13.447	0.76107	0.4906	9948
**Type x Line**	1	150.65	150.65	8.5266	0.0001	9944
**Section x Line**	1	26.922	26.922	1.5238	0.1796	9950
**Type x Section x Line**	1	11.113	11.113	0.629	0.5837	9931
**Residual**	533	9417.2	17.668			
**Total**	540	15268				

B. Ileum digesta

PERMANOVA table of results						
**Source**	**df**	**SS**	**MS**	**Pseudo-F**	**P(perm)**	**Unique perms**
**Line**	1	27.371	27.371	1.9859	0.1193	9949
**Residual**	179	2467.1	13.783			
**Total**	180	2494.4				

C. Ileum mucosa

PERMANOVA table of results						
**Source**	**df**	**SS**	**MS**	**Pseudo-F**	**P(perm)**	**Unique perms**
**Line**	1	41.369	41.369	1.6259	0.1463	9946
**Residual**	171	4351	25.444			
**Total**	172	4392.3				

D. Caecum digesta

PERMANOVA table of results						
**Source**	**df**	**SS**	**MS**	**Pseudo-F**	**P(perm)**	**Unique perms**
**Line**	1	77.404	77.404	6.7559	0.0015	9952
**Residual**	92	1054.1	11.457			
**Total**	93	1131.5				

E. Caecum mucosa

PERMANOVA table of results						
**Source**	**df**	**SS**	**MS**	**Pseudo-F**	**P(perm)**	**Unique perms**
**Line**	1	73.802	73.802	4.3467	0.0009	9945
**Residual**	91	1545.1	16.979			
**Total**	92	1618.9				
